# Prevalence of female sexual dysfunction among women with systemic lupus erythematosus: a systematic review and meta-analysis

**DOI:** 10.3389/fimmu.2026.1673871

**Published:** 2026-05-04

**Authors:** Xiaowei Dai, Hongxiang Ding, Dikai Mao, Jiaguo Huang

**Affiliations:** 1 Department of Reproductive Medicine Center, The Second Norman Bethune Hospital of Jilin University, Changchun, China; 2 Department of Urology, Affiliated Xiaoshan Hospital, Hangzhou Normal University, Hangzhou, China

**Keywords:** female sexual dysfunction, meta-analysis, prevalence, systematic review, systemic lupus erythematosus

## Abstract

**Background:**

The prevalence of female sexual dysfunction (FSD) among women with systemic lupus erythematosus (SLE) is inconsistent, and whether SLE is a risk factor for FSD among women remains controversial. Therefore, this study aimed to conduct a systematic review and meta-analysis to estimate the prevalence of FSD among women with SLE and further explore the association between SLE and FSD.

**Methods:**

Literature on the prevalence of FSD among women with SLE was retrieved from PubMed, Web of Science, Cochrane Library, and Embase databases from inception to July 1, 2025. The pooled prevalence was calculated using a random-effects model for the meta-analysis. The Cochran *Q* and *I*^2^ tests were employed to examine heterogeneity among the studies, while subgroup analyses and meta-regression were conducted to identify potential sources of heterogeneity.

**Results:**

This meta-analysis included 13 studies involving 1,511 women with SLE and 2,246 healthy controls. The pooled prevalence of FSD among women with SLE was 58.8% (95% confidence interval [CI], 0.461–0.716). Compared with the healthy control group, women with SLE had a significantly increased risk of FSD (odds ratio, 2.64; 95% CI, 1.27–5.47).

**Conclusion:**

This systematic review and meta-analysis demonstrated a high prevalence of FSD among women with SLE and a significant association between SLE and an increased risk of FSD.

**Systematic review registration:**

https://inplasy.com/inplasy-2025-7-0092/,identifier 202570092.

## Introduction

Systemic lupus erythematosus (SLE) is a potentially fatal chronic autoimmune disease that affects multiple systems in the body. During disease progression, it can involve all organs and tissues, with the potential to cause irreversible damage (1). The most commonly reported symptoms include muscle pain, fatigue, and mucocutaneous involvement, such as rash and photosensitivity (2). The disease is characterized by alternating periods of remission and flares. SLE predominantly affects women, with the highest incidence occurring during the childbearing years (2). Compared with the 1970s, the global prevalence of SLE has increased from 40/100,000 to 100/100,000 since the 2000s (3).

Female sexual dysfunction (FSD) is a common public health problem affecting women worldwide and is characterized by decreased or absent sexual desire, sexual arousal disorders, inadequate lubrication, sexual pain, inability to achieve sexual satisfaction, and difficulty achieving orgasm (4). The occurrence and progression of FSD involve complex pathophysiological processes, including anatomical, physiological, neurobiological, psychological, and hormonal factors (5–8). In the past, religious and cultural constraints have discouraged women from openly discussing sexual issues, leading to the neglect of female sexual medicine. FSD may have detrimental effects on women’s physical and mental health, as well as on marital relationships and overall quality of life (9). Recently, with societal and medical advancements, female sexual medicine has begun receiving increased attention.

The symptoms of SLE can affect sexual function in multiple ways. Physical and psychological constraints associated with the disease may negatively affect sexual life (10, 11). Although many studies have reported sexual dysfunction among women with SLE, the reported prevalence and risk estimates of FSD remain inconsistent (12–14). While most studies identify SLE as a risk factor for FSD, others report contradictory findings (12). To date, no systematic review or meta-analysis has comprehensively summarized the available data to accurately estimate the prevalence of FSD among women with SLE and determine whether SLE is a risk factor for FSD. Therefore, this study aimed to provide a quantitative synthesis by summarizing the global prevalence of FSD among women with SLE and investigating the association between SLE and FSD risk. This may help inform FSD prevention and treatment.

## Methods

### Search strategy

This systematic review and meta-analysis was conducted in accordance with the Preferred Reporting Items for Systematic Reviews and Meta-analyses (PRISMA) guidelines (15). To identify relevant literature on the prevalence of FSD among women with SLE, PubMed, Cochrane Library, Web of Science, and Embase were systematically searched for studies published from inception to July 1, 2025, without language restrictions. The search terms consisted of a combination of Medical Subject Headings (MeSH) and other related keywords. **Table 1** presents the PubMed search query, while the search strategies for the other databases are provided in the Supplementary File (**Supplementary Tables 1-3**). The reference lists of the included studies were manually screened to identify further relevant literature.

**Table 1:** Search query used for the PubMed database.

Search line	Search query
#1	((((((((((((((((((((((((((Sexual Dysfunction[Title/Abstract]) OR (Sexual Dysfunctions[Title/Abstract])) OR (Sexual Disorder[Title/Abstract])) OR (Sexual Disorders[Title/Abstract])) OR (Psychosexual Dysfunction[Title/Abstract])) OR (Psychosexual Dysfunctions[Title/Abstract])) OR (Psychosexual Disorder[Title/Abstract])) OR (Psychosexual Disorders[Title/Abstract])) OR (Sexual Desire Dysfunction[Title/Abstract])) OR (Sexual Desire Dysfunctions[Title/Abstract])) OR (Sexual Desire Disorder[Title/Abstract])) OR (Sexual Desire Disorders[Title/Abstract])) OR (Sexual Aversion Dysfunction[Title/Abstract])) OR (Sexual Aversion Dysfunctions[Title/Abstract])) OR (Sexual Aversion Disorder[Title/Abstract])) OR (Sexual Aversion Disorders[Title/Abstract])) OR (Orgasmic Dysfunction[Title/Abstract])) OR (Orgasmic Dysfunctions[Title/Abstract])) OR (Orgasmic Disorder[Title/Abstract])) OR (Orgasmic Disorders[Title/Abstract])) OR (Sexual Arousal Dysfunction[Title/Abstract])) OR (Sexual Arousal Dysfunctions[Title/Abstract])) OR (Sexual Arousal Disorder[Title/Abstract])) OR (Sexual Arousal Disorders[Title/Abstract])) OR (Frigidity[Title/Abstract])) OR (Dyspareunia[Title/Abstract])) OR (Vaginismus[Title/Abstract])
#2	(“Lupus Erythematosus, Systemic”[Mesh]) OR (((((Lupus Erythematosus Disseminatus[Title/Abstract]) OR (Systemic Lupus Erythematosus[Title/Abstract])) OR (Libman-Sacks Disease[Title/Abstract])) OR (Disease, Libman-Sacks[Title/Abstract])) OR (Libman Sacks Disease[Title/Abstract]))
#3	#1 AND #2

MeSH, Medical Subject Headings.

### Study selection and data extraction

Studies were deemed eligible if they reported the prevalence of FSD among women with SLE. Studies were excluded based on the following criteria: (1) Case reports, editorials, letters to the editor, conference abstracts, or reviews; (2) duplicate publications or studies reporting the same sample; (3) studies lacking extractable information on the main outcomes.

Two independent investigators (XD and HD) initially screened the titles and abstracts of all retrieved records. The full texts of potentially eligible studies were then assessed for final inclusion. Non-English articles were translated using Google Translate, and the translations were then reviewed and verified by the professionals. In cases of duplicate or overlapping publications, the study with the most recent and complete data was selected. Disagreements were resolved through consultation with a third investigator (DM).

Data were independently extracted and verified for accuracy by two investigators (XD and HD), and the results were recorded in a Microsoft Excel spreadsheet (Microsoft Corporation, Redmond, WA). The following data were collected: First author’s name, year of publication, study region (country), study design, mean or median age, assessment measure of FSD, sample size, number of women with FSD, sample size of healthy controls without SLE (if available), and number of controls with FSD (if available).

### Quality assessment

The quality of the included studies was assessed using the Agency for Healthcare Research and Quality (AHRQ) tool for cross-sectional studies (16) and the Newcastle-Ottawa Scale (NOS) tool for case-control studies (17). Both quality assessment tools are available at: https://www.ncbi.nlm.nih.gov/books/NBK35156/. Two investigators (XD and HD) independently performed quality assessments. Higher scores indicate better methodological quality. AHRQ scores range from 0 to 11, with studies classified as high (scores 8–11), moderate (scores 4–7), or low (scores 0–3) quality. NOS scores range from 0 to 9, with studies classified as high (scores 7–9), moderate (scores 4–6), or low (scores 0–3) quality.

### Study outcomes and statistical analysis

Meta-analysis was performed using Stata software (version 16.0; StataCorp). The pooled prevalence of FSD was calculated using 95% confidence interval (CI). Heterogeneity among the included studies was assessed using the Cochran *Q* test, and *I*^2^ statistic was used to quantify the degree of heterogeneity (18). Heterogeneity was classified as low (< 25%), moderate (25%–75%), or high (> 75%).

A fixed-effects model was applied when *I*^2^ <50% and/or *P* > 0.05; otherwise, a random-effects model was used (19). In cases of high heterogeneity, potential sources were explored through subgroup analyses and meta-regression. Sensitivity analysis was conducted to evaluate the influence of individual studies on the overall results (20). Publication bias was assessed using Egger’s test (19, 21). Logistic regression results were reported as odds ratio (OR) with 95% CI. *P* < 0.05 was considered statistically significant.

## Results

**Figure 1:**
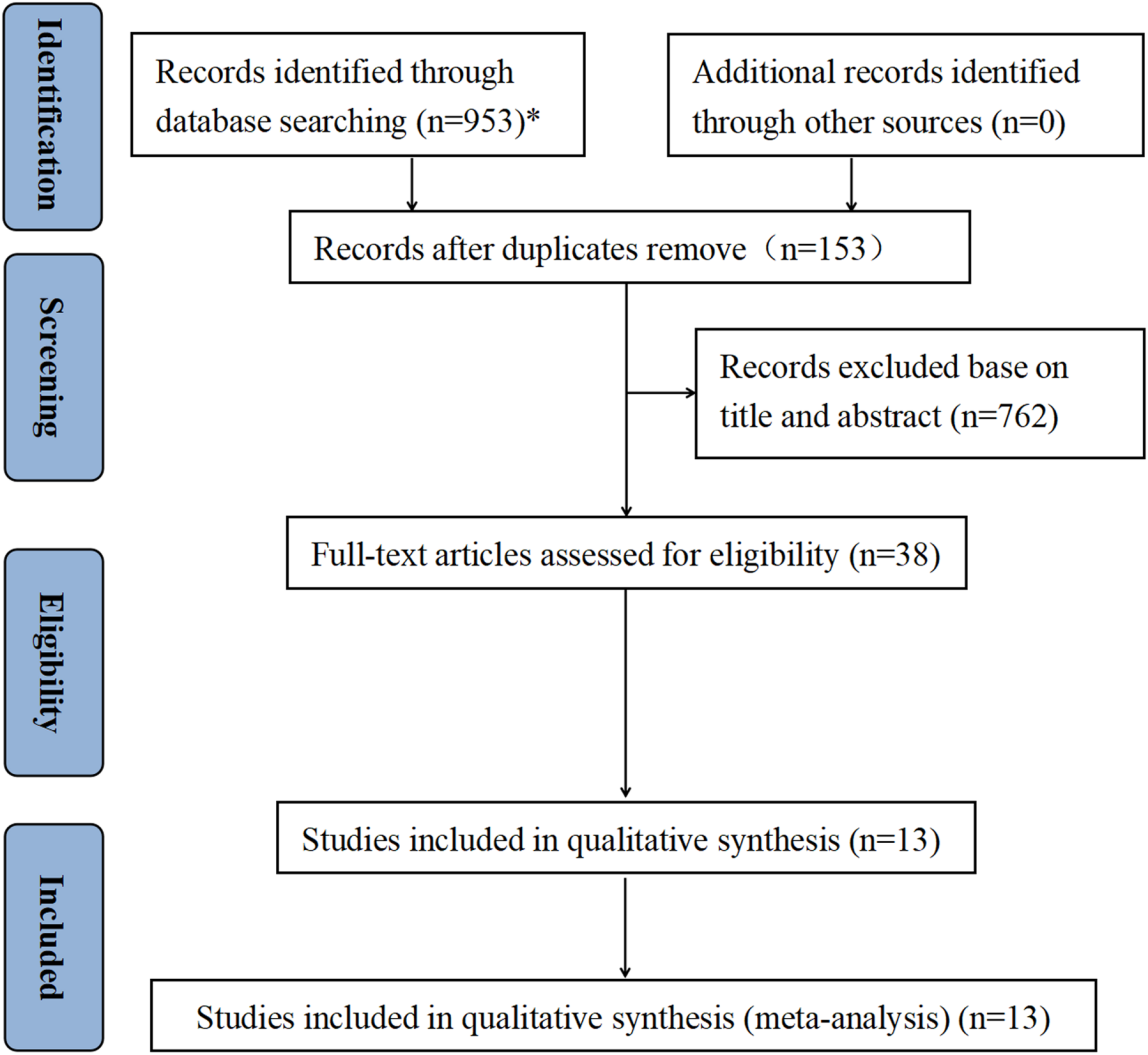
PRISMA flow diagram illustrating the study selection process. *Databases searched, and the number of records identified were: PubMed (n = 45), Web of Science (n = 824), Cochrane Library (n = 7), and Embase (n = 77).

A total of 953 publications were identified through database searches: PubMed (n = 45), Web of Science (n = 824), the Cochrane Library (n = 7), and Embase (n = 77). After removing 153 duplicate articles using EndNote and manual checking, the titles and abstracts of the remaining records were screened, resulting in 38 studies that were selected for full-text review. Finally, 13 studies met the inclusion criteria and were included in the meta-analysis (**Figure 1**) (10, 12–14, 22–30).

The 13 studies included 1,511 women with SLE and 2,246 healthy controls. As presented in **Table 2**, the mean age of the participants ranged from 16.7 ± 1.94 to 54.7 ± 14.2 years. The reported prevalence of FSD among women with SLE ranged from 22% to 85.9%. Most studies (12/13) used the Female Sexual Function Index (FSFI) questionnaire to assess FSD, while one study relied on clinical history. Geographically, 6 studies were conducted in Asia, 3 in Europe, 3 in the Americas, and 1 in Africa. Regarding study design, 5 were cross-sectional studies, and 8 were case-control studies; 12 were single-center studies and 1 was multicenter study. AHRQ scores for the cross-sectional studies ranged from 7 to 9, while NOS scores for the case-control studies ranged from 6 to 8. Overall, 9 were classified as high quality, and 4 as moderate quality (Supplementary File **Supplementary Tables 4**-**5**).

**Table 2:** Summary of characteristics of studies included in the meta-analysis.

Study	Region	Study design	Number of study centers	Mean age (years)	Measure	SLE		Healthy controls		OR	LCI	UCI	Quality assessment	Score
						Sample size	FSD cases	Sample size	FSD cases					
Anyfanti, P. 2013 (22)	Greece	Cross-sectional	Single-center	54.7 ± 14.2	FSFI	46	31	**-**	**-**	**-**	**-**	**-**	AHRQ	8
Da Silva, C. A. A. 2009 (23)	Brazil	Case-control	Single-center	16.7 ± 1.94	Clinical history	52	30	52	12	4.545	1.947	10.612	NOS	6
Dag, A. 2024 (24)	Turkey	Case-control	Single-center	41.3 ± 9.1	FSFI	49	37	52	22	4.205	1.793	9.861	NOS	8
Dorgham, D. 2020 (12)	Egypt	Case-control	Single-center	32.5 ± 5.6	FSFI	94	73	98	81	0.73	0.357	1.489	NOS	8
Ferreira, C. C. 2013 (13)	Brazil	Cross-sectional	Multicenter	36.1 ± 10.1	FSFI	82	18	**-**	**-**	–	–	–	AHRQ	7
Garcia Morales, M. 2013 (14)	Spain	Case-control	Single-center	39.03 ± 10.83	FSFI	61	28	52	15	2.093	0.956	4.581	NOS	8
Moghadam, Z. B. 2019 (25)	Iran	Case-control	Single-center	37.64 ± 7.96	FSFI	170	146	170	45	16.898	9.75	29.288	NOS	6
Pinto, B. 2019 (26)	India	Cross-sectional	Single-center	34 ± 6.8	FSFI	112	67	**-**	**-**	–	–	–	AHRQ	8
Serna-Peña, G. 2021 (27)	Mexico	Case-control	Single-center	36.3 ± 11.6	FSFI	65	18	121	27	1.333	0.668	2.663	NOS	6
Tseng, J. 2011 (28)	Taiwan	Case-control	Single-center	37.5 ± 10.2	FSFI	279	85	1580	408	1.259	0.952	1.663	NOS	7
Xia, X. Y. 2024 (29)	China	Cross-sectional	Single-center	35.26 ± 6.28	FSFI	293	173	**-**	**-**	–	–	–	AHRQ	9
Yi, Q. 2016 (30)	China	Cross-sectional	Single-center	38.6 ± 9	FSFI	80	61	**-**	**-**	–	–	–	AHRQ	8
Zhang, L. 2022 (10)	China	Case-control	Single-center	43.65 ± 7.13	FSFI	128	101	121	68	2.916	1.672	5.085	NOS	7

AHRQ, Agency for Healthcare Research and Quality; CI, confidence interval; FSD, female sexual dysfunction; FSFI, Female Sexual Function Index; NOS, Newcastle-Ottawa Scale; UCI, upper confidence interval; OR, odds ratio; SLE, systemic lupus erythematosus.

**Figure 2:**
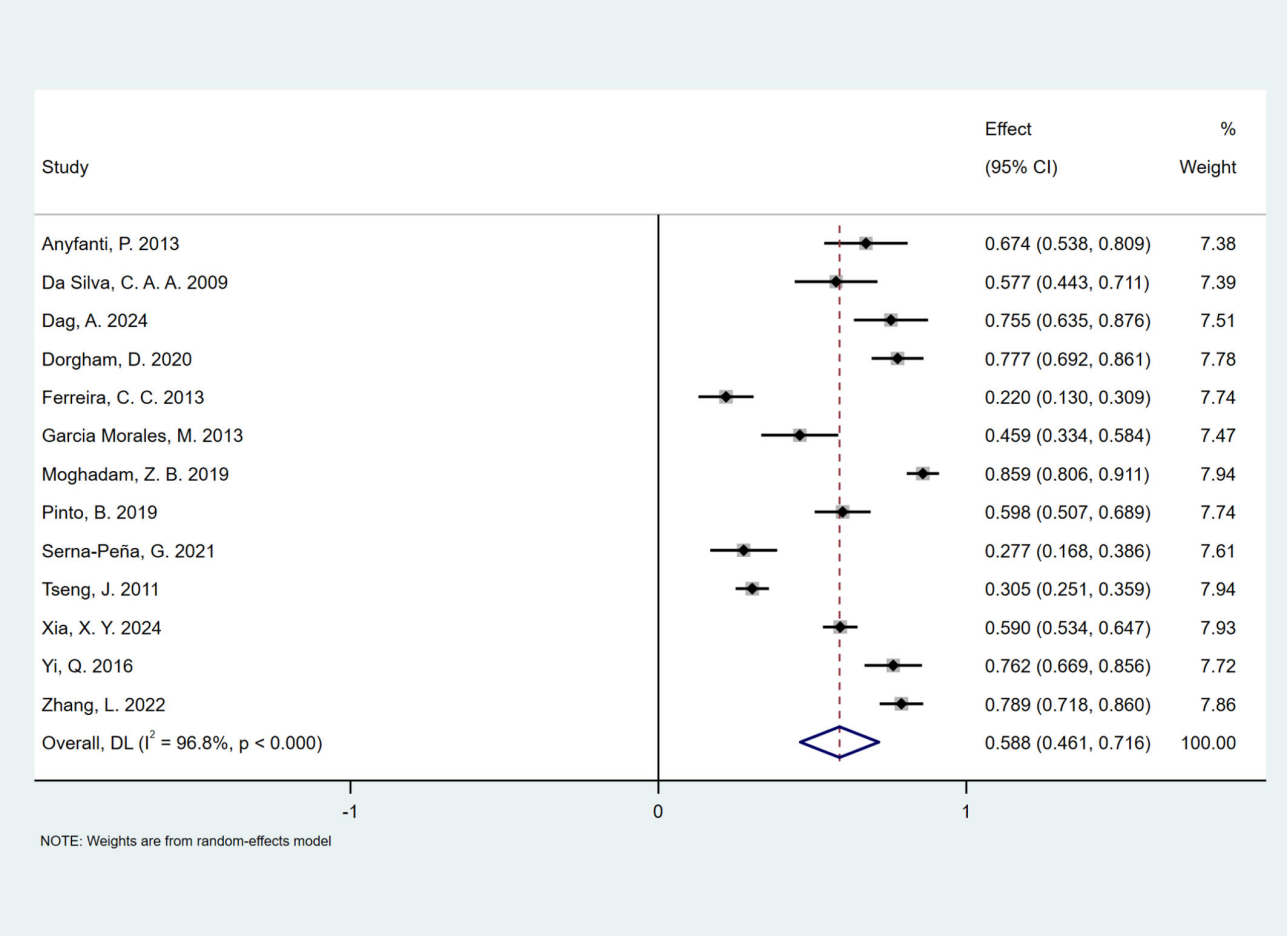
Forest plot demonstrating the pooled prevalence of FSD among women with SLE.

The pooled prevalence of FSD among women with SLE was 58.8% (95% CI, 0.461–0.716). Due to high heterogeneity (*I*^2^ = 96.8%, *P* < 0.001; Cochran *Q* test *P* < 0.001), a random-effects model was applied. **Figure 2** presents a forest plot of the meta-analysis results. In the included studies, the prevalence of FSD ranged from 22% to 85.9%, with study weights ranging from 7.38% to 7.94%.

Subgroup analyses were conducted according to the study region, design, and quality to evaluate the differences in the FSD prevalence (**Table 3**). When grouped by region, the prevalence of FSD was 77.7% in Africa, 65.8% in Asia, 61.6% in Europe, and 34.9% in the Americas. Based on the study design, the prevalence was 56.8% in cross-sectional studies and 60.9% in case-control studies. Regarding study quality, the prevalence of FSD was 63.7% in the high-quality group and 48.9% in the moderate-quality group. Subgroup analyses for the number of study centers and FSD assessment measures were not performed due to the limited number of studies. A meta-regression analysis was conducted to assess the effects of age, sample size, and publication year on FSD prevalence. However, no significant associations were identified between FSD prevalence and any covariates (**Table 4**).

**Table 3:** Subgroup analysis of FSD prevalence according to different items.

Subgroups	Items	No. of studies	Prevalence, % (95% CI)	Heterogeneity test		Heterogeneity between subgroups
				*P* value	*I*^2^, %	*P* value
Study region	Asia	6	65.8 (48.6–81.2)	<0.001	96.8	0.003
	Europe	3	61.6 (40.1–80.2)	<0.001	85.8	
	The Americas	3	34.9 (15.7–57.0)	<0.001	90.0	
	Africa	1	77.7 (68.7–85.6)	–	–	
Study design	Cross-sectional	5	56.8 (40.2–72.6)	<0.001	93.3	0.754
	Case-control	8	60.9 (41.2–78.9)	<0.001	97.0	
Study quality	High quality	9	63.7 (50.5–75.9)	<0.001	94.8	0.453
	Moderate quality	4	48.9 (15.6–82.8)	<0.001	97.8	

CI, confidence interval.

**Table 4:** Meta-regression analysis for the effect of each moderator on the prevalence of FSD.

Moderator	No. of studies	Regression coefficient	95% CI	P value	τ ^2^	*I*^2^, %
Age	13	0.0192	-0.06–0.10	0.595	0.9329	95.27
Simple size	13	-0.0009	-0.01–0.01	0.790	0.9559	94.99
Study publication year	13	0.0807	-0.04–0.20	0.153	0.7777	93.58

CI, confidence interval.

**Figure 3:**
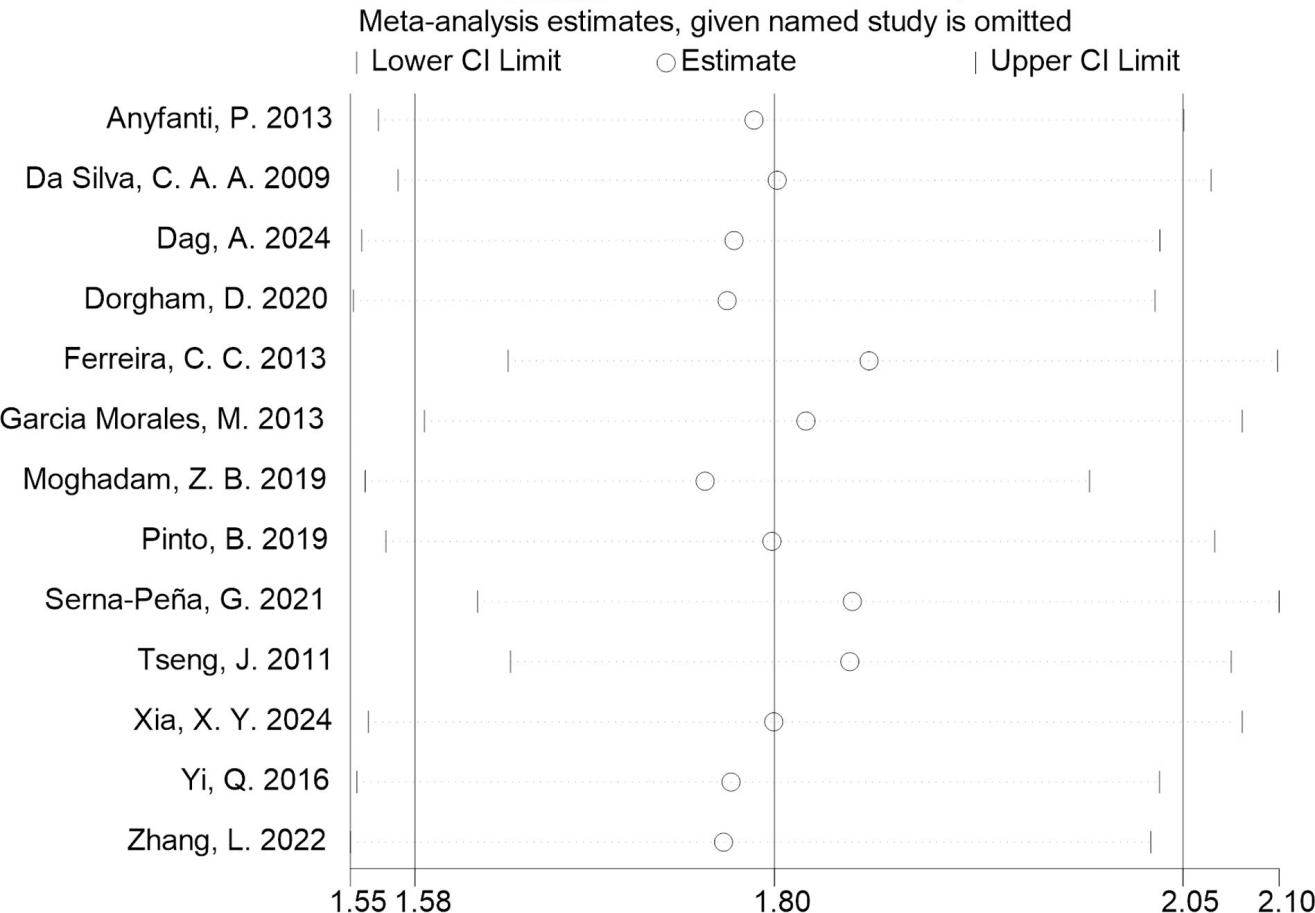
Leave-one-out sensitivity analysis of the pooled prevalence. By sequentially excluding each individual study, sensitivity analysis showed that no particular study could significantly influence the result, indicating that the meta-analysis findings were stable.

**Figure 4:**
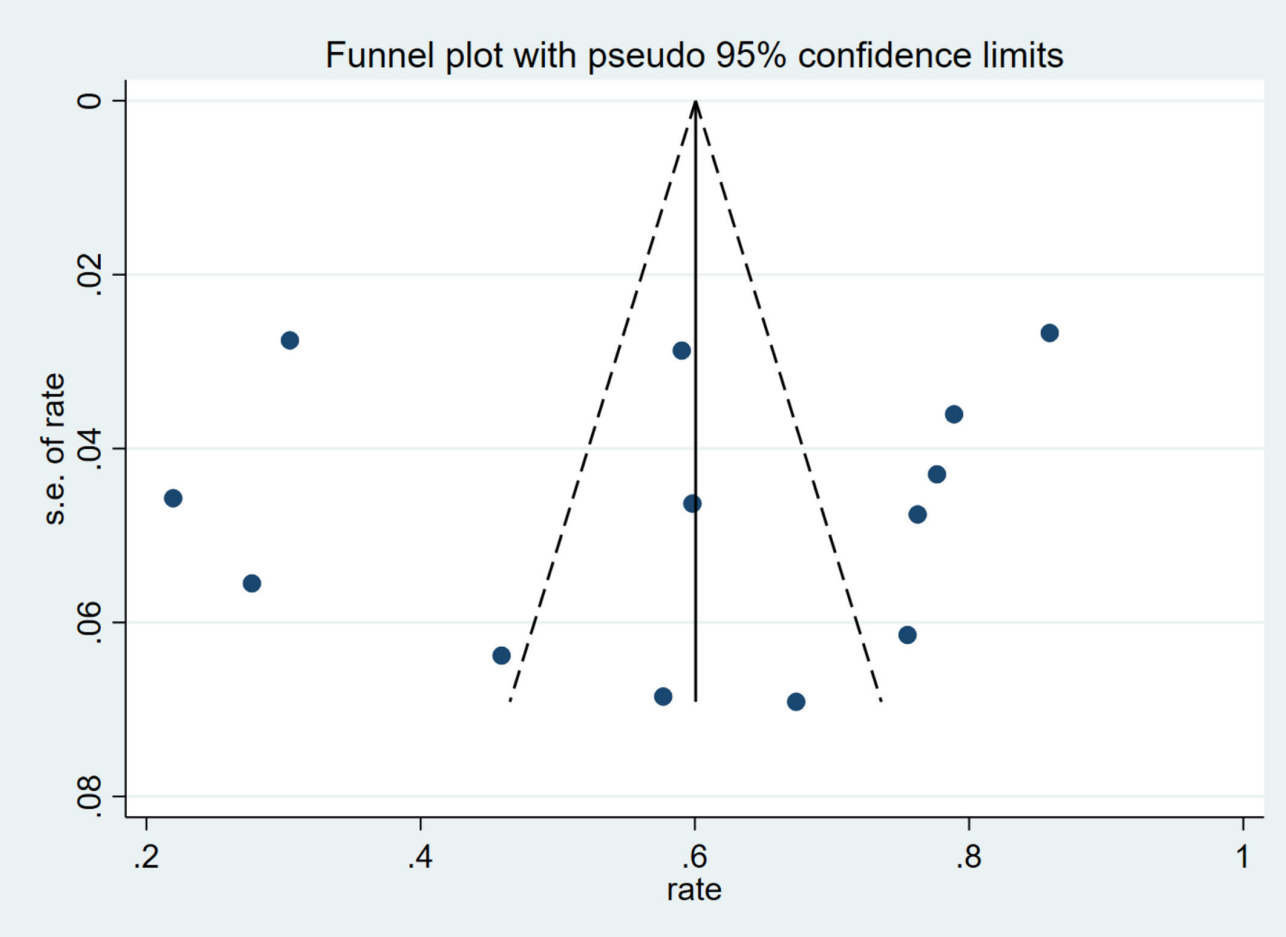
Funnel plot was roughly symmetric, indicating no significant publication bias among included studies.

A sensitivity analysis was performed using the leave-one-out method. The results depicted in **Figure 3**, indicated that the meta-analysis findings were stable. Based on the funnel plot (**Figure 4**) and Egger’s test results (*P* = 0.786), no publication bias was observed.

**Figure 5:**
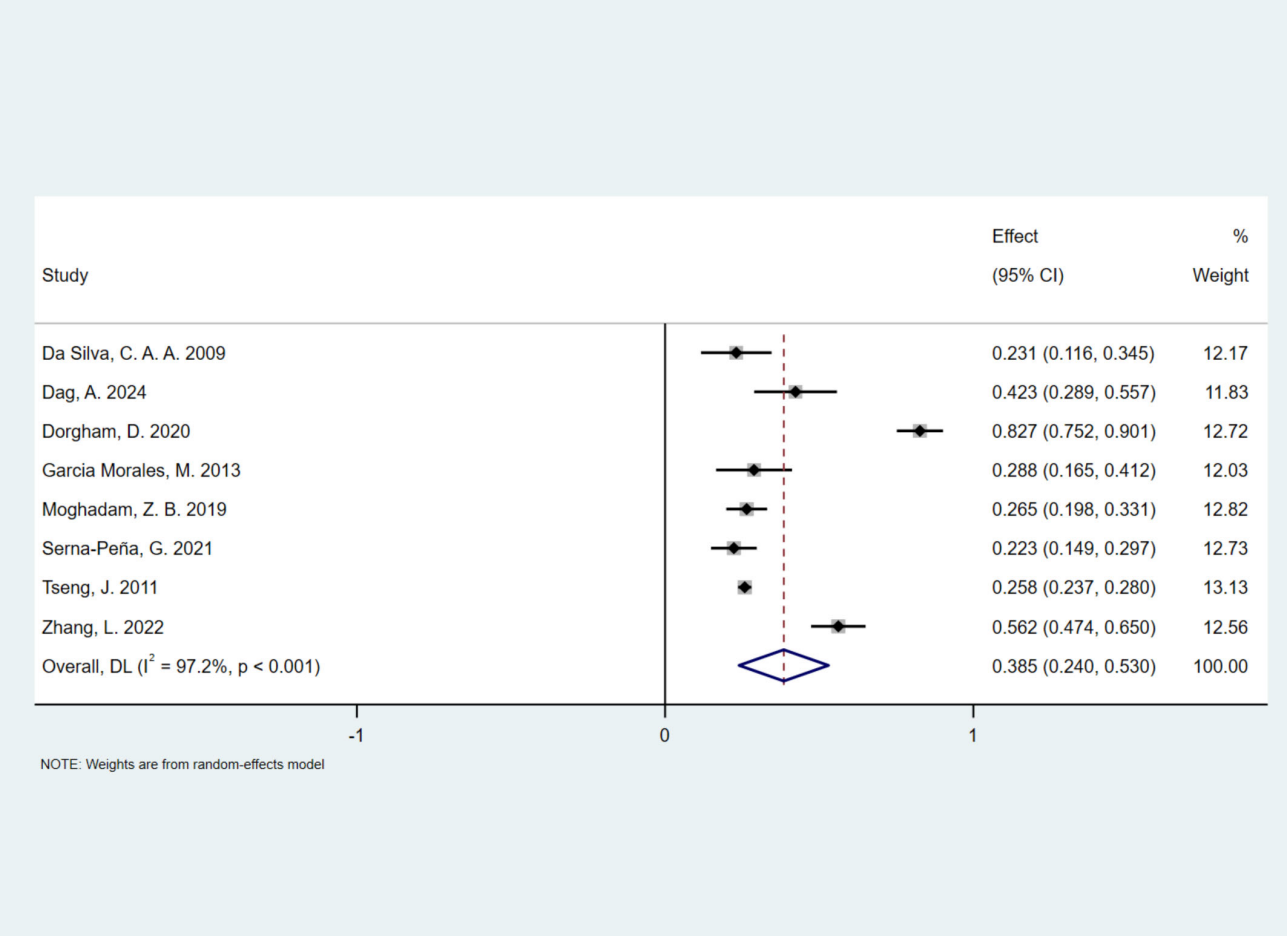
Forest plot demonstrating the pooled prevalence of FSD among healthy controls.

**Figure 6:**
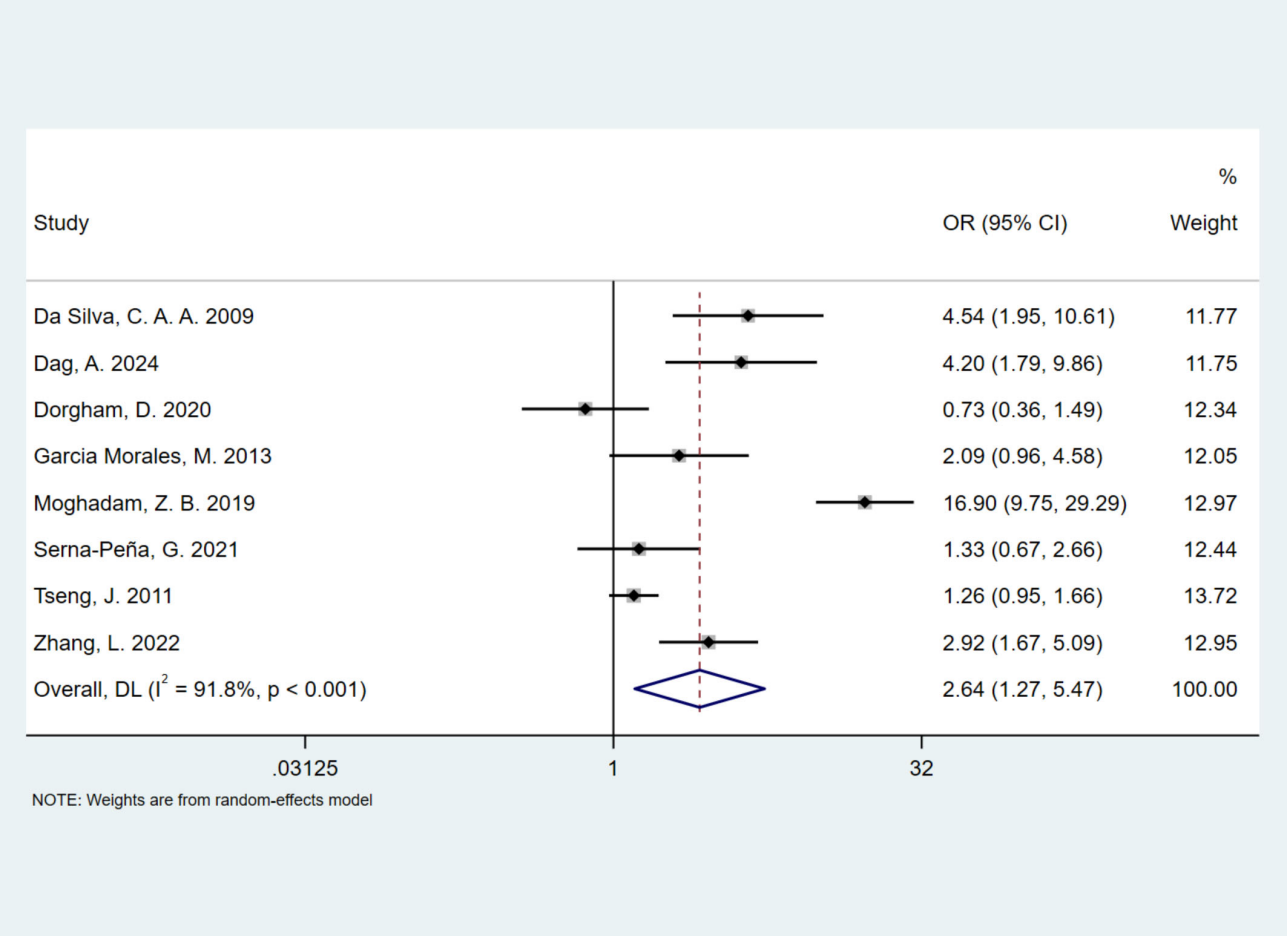
Forest plot depicting the OR of FSD among women with SLE as compared with healthy controls.

The pooled prevalence of FSD in healthy controls was 38.5% (95% CI, 0.240–0.530), which was significantly lower than that in the SLE group. Due to high heterogeneity (*I*^2^ = 97.2%, *P* < 0.001; Cochran *Q* test *P* < 0.001), a random-effects model was applied. **Figure 5** presents a forest plot of the meta-analysis results. **Figure 6** illustrates the association between SLE and FSD risk. Women with SLE had a significantly higher risk of FSD (OR, 2.64; 95% CI, 1.27–5.47) compared with healthy controls.

## Discussion

The main finding of this study is that the pooled prevalence of FSD among women with SLE is 58.8% (95% CI, 0.461–0.716). For comparison, a previous meta-analysis reported a prevalence of 49.1% among women with rheumatoid arthritis (31). These findings suggest that women with autoimmune diseases are generally at a higher risk of sexual dysfunction, highlighting the need for greater attention from both society and the medical community. In clinical practice, it is important to closely monitor sexual function and satisfaction among women with SLE. We recommend comprehensive assessments and further epidemiological research to explore the shared pathophysiological mechanisms between SLE and FSD. Such efforts are essential for developing targeted prevention and intervention strategies, with the ultimate goal of reducing the prevalence of FSD among women with SLE and improving their quality of life.

Research has found that women with SLE have a higher prevalence of FSD and that this condition exhibits significant heterogeneity. A statistically significant difference was observed in the pooled prevalence estimates across regions (*P* = 0.003), with the highest prevalence reported among African women with SLE. Previous studies have also demonstrated that the prevalence of FSD varies among different ethnic groups. Black women tend to report higher rates of low sexual desire and experience less sexual pleasure, whereas White women are more likely to experience sexual pain (32). Additionally, the prevalence of SLE is higher among Black populations (33, 34). Variations in race, environment, socio-economic status, and healthcare access across regions may contribute to these disparities (33). For instance, countries near the equator are exposed to higher levels of ultraviolet radiation, which has been hypothesized as an environmental trigger for SLE (35).

Differences in gene-environment interactions may contribute to the higher incidence and prevalence of SLE among Black populations migrating from Africa. This hypothesis is currently being investigated in the Gullah population in South Carolina in comparison with individuals from their ancestral origin in Sierra Leone (36, 37). Conversely, lower diagnosis rates in some regions may be due to underdeveloped local economies and limited healthcare resources. Accordingly, the study region may represent an important explanatory variable for heterogeneity in prevalence estimates. Most prevalence estimates of FSD in women with SLE have been reported from Asia. As few studies have been conducted in other parts of the world, these results should be interpreted with caution. Furthermore, heterogeneity within each regional subgroup remained high, indicating substantial variation even within the same region. This suggests that other factors, such as FSD assessment measures, sample size, or number of study centers, may influence the findings. However, due to the limited number of studies in each region, further subgroup analyses could not be performed.

Previous studies have reported that the prevalence of FSD in the general population ranges from 22.6% to 49% (42–44). However, it remains unclear whether women with SLE are more susceptible to FSD than the general population, and whether SLE independently increases the risk of FSD. According to the consensus of the 4th International Consultation for Sexual Medicine in 2015, 40%–50% of women experience at least one sexual dysfunction, regardless of age (45). In our study, the prevalence of FSD in the healthy control group was 38.5%. Compared with healthy controls, women with SLE had a 2.64-fold higher prevalence of FSD, further supporting SLE as a risk factor for FSD. Among women with SLE, age, menstrual status, depression, fatigue, disease activity, and disease duration were identified as risk factors for FSD (10, 12, 28, 29). In the general population, the prevalence of FSD seems to increase with age (38). Women experience a gradual decline in estrogen levels during perimenopause, which becomes more pronounced after menopause (39). As estrogen levels decrease, sexual desire and arousal often decline, vaginal atrophy can impair lubrication and cause painful intercourse, and overall sexual satisfaction and the ability to achieve orgasm may be affected (40, 41).

Patients with SLE often experience pain, fatigue, and functional limitations due to recurrent symptoms, which can result in loss of work capacity and the need for long-term glucocorticoid therapy. These factors may contribute to negative psychological outcomes, including anxiety and depression (46, 47). In a survey of 147 patients with SLE, Bultink et al. reported that fatigue was a common and persistent symptom (48). Although the direct impact of glucocorticoids on sexual function in women with SLE remains unclear, obesity and changes in physical appearance frequently cause substantial psychological stress, leading to anxiety, depression, and increased risk of FSD (49). These findings indicate that a majority of women with SLE experience FSD, which can profoundly affect their personal, familial, and social well-being. Therefore, clinical practice should prioritize targeted interventions addressing key risk factors, alongside education and support for patients and their families to improve sexual health and overall quality of life.

Due to religious and cultural constraints, many women are hesitant or unwilling to openly discuss sexual issues, limiting the availability of data on FSD, particularly among women with SLE. A major strength of our study is the comprehensive inclusion of relevant studies on the prevalence of FSD in this population. We systematically searched four databases to identify eligible articles. Additionally, most of the included studies were rated as high or moderate quality, providing robust evidence for the prevalence of FSD among women with SLE. Finally, we conducted a random-effects meta-analysis and sensitivity analysis, both of which confirmed the stability of the pooled prevalence estimates.

This study has several limitations. First, regarding publication bias, most available literature consists of observational studies, which are inherently prone to bias. However, our sensitivity analysis indicated that the meta-analysis results were stable, and data extraction was independently performed by two researchers with verification of accuracy. Second, most included studies were single-center with relatively small sample sizes, which may have reduced the reliability of the results due to limited statistical power. Third, the results of the subgroup analysis based on study region should be interpreted with caution, given the limited number of studies and potential confounding factors. Consequently, further investigation of the association between FSD and SLE, as well as the identification of risk factors for FSD among women with SLE, requires prospective, multicenter, and long-term studies.

## Conclusion

This study highlights that FSD is a significant health concern for women with SLE. Based on the findings of this systematic review and meta-analysis, the pooled prevalence of FSD among women with SLE was 58.8%, and SLE was significantly associated with an increased risk of FSD. These results suggest that rheumatologists and policymakers should consider FSD in clinical guidelines and treatment pathways and pay greater attention to patient care.

## Data Availability

The original contributions presented in the study are included in the article/**Supplementary Material**. Further inquiries can be directed to the corresponding author.

## Supplementary material

Supplementary Table 1 Search query used for the Cochrane Library database.

Supplementary Table 2 Search query used for the Web of Science database.

Supplementary Table 3 Search query used for the Embase database.

Supplementary Table 4 Quality assessment of cross-sectional studies using the Agency for Healthcare Research and Quality (AHRQ) methodology checklist.

Supplementary Table 5 Quality assessment of case–control studies using the Newcastle-Ottawa Scale (NOS) methodology checklist.
