# Supplementary material for: Prevalence of female sexual dysfunction among women with systemic lupus erythematosus: a systematic review and meta-analysis
Source: Front Immunol. 2026 May 4;17:1673871. doi: 10.3389/fimmu.2026.1673871 (PMC13180918; doi:10.3389/fimmu.2026.1673871)
Supplement: Supplementary Table 1 — Search query used for the Cochrane Library database. [file DataSheet1.pdf]

Prevalence of Sexual Dysfunction among Females with Systemic Lupus Erythematosus: A Systematic Review and Meta-analysis

Xiaowei Dai[1], Hongxiang Ding[2], Dikai Mao[2], Jiaguo Huang[2*]

[1]Department of Reproductive Medicine Center, The Second Norman Bethune Hospital of Jilin University, Changchun, China;

[2]Department of Urology, Affiliated Xiaoshan Hospital, Hangzhou Normal University, Hangzhou, China.

*** Correspondence:**

Jiaguo Huang

13735526508@163.com

Table S1 Search query used for the Cochrane Library database.

Search line	Search query
#1	(Sexual Dysfunction):ab,ti,kw OR(Sexual Dysfunctions):ab,ti,kw OR(Sexual Disorder):ab,ti,kw OR(Sexual Disorders):ab,ti,kw OR(Psychosexual Dysfunction):ab,ti,kw OR(Psychosexual Dysfunctions):ab,ti,kw OR(Psychosexual Disorder):ab,ti,kw OR(Psychosexual Disorders):ab,ti,kw OR(Sexual Desire Dysfunction):ab,ti,kw OR(Sexual Desire Dysfunctions):ab,ti,kw OR(Sexual Desire Disorder):ab,ti,kw OR(Sexual Desire Disorders):ab,ti,kw OR(Sexual Aversion Dysfunction):ab,ti,kw OR(Sexual Aversion Dysfunctions):ab,ti,kw OR(Sexual Aversion Disorder):ab,ti,kw OR(Sexual Aversion Disorders):ab,ti,kw OR(Orgasmic Dysfunction):ab,ti,kw OR(Orgasmic Dysfunctions):ab,ti,kw OR(Orgasmic Disorder):ab,ti,kw OR(Orgasmic Disorders):ab,ti,kw OR(Sexual Arousal Dysfunction):ab,ti,kw OR(Sexual Arousal Dysfunctions):ab,ti,kw OR(Sexual Arousal Disorder):ab,ti,kw OR(Sexual Arousal Disorders):ab,ti,kw OR(Frigidity):ab,ti,kw OR(Dyspareunia):ab,ti,kw OR(Vaginismus):ab,ti,kw
#2	(Lupus Erythematosus Disseminatus):ab,ti,kw OR(Systemic Lupus Erythematosus):ab,ti,kw OR(Libman-Sacks Disease):ab,ti,kw OR(Disease, Libman-Sacks):ab,ti,kw OR(Libman Sacks Disease):ab,ti,kw
#3	#1 AND #2

Table S2 Search query used for the Web of Science database.

Search line	Search query
#1	Sexual Dysfunction OR Sexual Dysfunctions OR Sexual Disorder OR Sexual Disorders OR Psychosexual Dysfunction OR Psychosexual Dysfunctions OR Psychosexual Disorder OR Psychosexual Disorders OR Sexual Desire Dysfunction OR Sexual Desire Dysfunctions OR Sexual Desire Disorder OR Sexual Desire Disorders OR Sexual Aversion Dysfunction OR Sexual Aversion Dysfunctions OR Sexual Aversion Disorder OR Sexual Aversion Disorders OR Orgasmic Dysfunction OR Orgasmic Dysfunctions OR Orgasmic Disorder OR Orgasmic Disorders OR Sexual Arousal Dysfunction OR Sexual Arousal Dysfunctions OR Sexual Arousal Disorder OR Sexual Arousal Disorders OR Frigidity OR Dyspareunia OR Vaginismus
#2	Lupus Erythematosus Disseminatus OR Systemic Lupus Erythematosus OR Libman-Sacks Disease OR Disease, Libman-Sacks OR Libman Sacks Disease
#3	#1 AND #2

Table S3 Search query used for the Embase database.

Search line	Search query
#1	'Sexual Dysfunction':ab,ti OR 'Sexual Dysfunctions':ab,ti OR 'Sexual Disorder':ab,ti OR 'Sexual Disorders':ab,ti OR 'Psychosexual Dysfunction':ab,ti OR 'Psychosexual Dysfunctions':ab,ti OR 'Psychosexual Disorder':ab,ti OR 'Psychosexual Disorders':ab,ti OR 'Sexual Desire Dysfunction':ab,ti OR 'Sexual Desire Dysfunctions':ab,ti OR 'Sexual Desire Disorder':ab,ti OR 'Sexual Desire Disorders':ab,ti OR 'Sexual Aversion Dysfunction':ab,ti OR 'Sexual Aversion Dysfunctions':ab,ti OR 'Sexual Aversion Disorder':ab,ti OR 'Sexual Aversion Disorders':ab,ti OR 'Orgasmic Dysfunction':ab,ti OR 'Orgasmic Dysfunctions':ab,ti OR 'Orgasmic Disorder':ab,ti OR 'Orgasmic Disorders':ab,ti OR 'Sexual Arousal Dysfunction':ab,ti OR 'Sexual Arousal Dysfunctions':ab,ti OR 'Sexual Arousal Disorder':ab,ti OR 'Sexual Arousal Disorders':ab,ti OR 'Frigidity':ab,ti OR 'Dyspareunia':ab,ti OR 'Vaginismus':ab,ti
#2	'Lupus Erythematosus Disseminatus':ab,ti OR 'Systemic Lupus Erythematosus':ab,ti OR 'Libman-Sacks Disease':ab,ti OR 'Disease, Libman-Sacks':ab,ti OR 'Libman Sacks Disease':ab,ti
#3	#1 AND #2

Table S4 Quality assessment of cross-sectional studies using the Agency for Healthcare Research and Quality (AHRQ) methodology checklist

Study	①	②	③	④	⑤	⑥	⑦	⑧	⑨	⑩	⑪	Total
Anyfanti, P. 2013	1	1	1	1	0	0	1	1	1	1	0	8
Ferreira, C. C. 2013	1	1	0	1	0	0	1	1	1	1	0	7
Pinto, B. 2019	1	1	0	1	0	1	1	1	1	1	0	8
Xia, X. Y. 2024	1	1	1	0	1	1	1	1	1	1	0	9
Yi, Q. 2016	1	1	1	0	1	1	1	1	0	1	0	8

Note: ① Define the source of information (survey, record review); ② List the inclusion and exclusion criteria for exposed and unexposed subjects (cases and controls) or refer to previous publications; ③ Indicate the time taken to identify patients; ④ Indicate whether or not subjects were consecutive if not population-based; ⑤ Indicate whether the evaluators of the subjective components of the study were masked to the other aspects of the status of the participants; ⑥ Describe any assessments undertaken for quality assurance purposes (e.g., test/retest of primary outcome measurements); ⑦ Explain any patient exclusions from the analysis; ⑧ Describe how confounding was assessed and/or controlled; ⑨ If applicable, explain how missing data were handled in the analysis; ⑩ Summarize patient response rates and completeness of data collection; ⑪ Clarify what follow-up, if any, was expected and the percentage of patients for which incomplete data or follow-up was obtained.

Table S5 Quality assessment of case–control studies using the Newcastle-Ottawa Scale (NOS) methodology checklist

Study	Selection				Comparability	Exposure/outcome			Total
	①	②	③	④	⑤	⑥	⑦	⑧	
Da Silva, C. A. A. 2009	1	0	1	1	1	1	1	0	6
Dag, A. 2024	1	1	1	1	2	1	1	0	8
Dorgham, D. 2020	1	1	0	1	2	1	1	1	8
Garcia Morales, M. 2013	1	1	0	1	2	1	1	1	8
Moghadam, Z. B. 2019	1	0	1	1	1	1	1	0	6
Serna-Peña, G. 2021	1	1	1	0	1	1	1	0	6
Tseng, J. 2011	1	1	0	1	1	1	1	1	7
Zhang, L. 2022	1	1	1	1	1	1	1	0	7

Note: ① Is the case definition adequate; ② Representativeness of the cases; ③ Selection of controls; ④ Definition of controls; ⑤ Comparability of cases and controls on the basis of the design or analysis (a maximum of two sores can be give for Comparability); ⑥ Ascertainment of exposure; ⑦ Same method of ascertainment for cases and controls; ⑧ Nonresponse rate.
